# Efficacy and Safety of Upadacitinib and Vedolizumab Combination for Refractory Orofacial Granulomatosis Associated with Panenteric Crohn’s Disease: A Case Report

**DOI:** 10.3390/reports8020037

**Published:** 2025-03-23

**Authors:** Michele Campigotto, Francesca Priotto, Cinzia Francesca Tonello, Fabio Monica, Saveria Lory Crocè

**Affiliations:** 1 Gastroenterology and Digestive Endoscopy Unit, Ca’Foncello Hospital, ULSS2 Marca Trevigiana, 31100 Treviso, Italy; 2 Department of Medicine, Surgery and Health Sciences, University of Trieste, 34127 Trieste, Italy; francypriotto@gmail.com (F.P.); lcroce@units.it (S.L.C.); 3 Gastroenterology and Digestive Endoscopy Unit, Cattinara University Hospital, 34127 Trieste, Italy; cinziafrancesca.tonello@asugi.sanita.fvg.it (C.F.T.); fabio.monica@asugi.sanita.fvg.it (F.M.); 4 Liver Clinic, Maggiore University Hospital, 34127 Trieste, Italy

**Keywords:** orofacial granulomatosis, inflammatory bowel disease, Crohn’s disease, Upadacitinib, JAK inhibitors

## Abstract

**Background and Clinical Significance**: Orofacial granulomatosis is a rare but often disabling condition potentially associated with inflammatory bowel disease (IBD). Pathogenesis is not well understood, and no disease-specific approved treatment exists to date. **Case Presentation**: A 26-year-old woman with pan-enteric Crohn’s disease developed buccal swelling and deep oral ulcers histologically confirmed as associated orofacial granulomatosis. Multiple therapies were attempted during her life, including systemic steroids and immunomodulator drugs as Thalidomide, Adalimumab, and Ustekinumab in combination with topical steroid injections and Cyclosporin application, with no or minimal benefit. Only Infliximab showed good efficacy, but it was suspended due to side effects. Following secondary loss of response to Ustekinumab, compassionate treatment with Upadacitinib, a recently developed oral Jak-1 selective inhibitor, resulted in the complete resolution of the oral ulcers. Moreover, after the 12-week induction phase and the transition to 30 mg/daily maintenance dosage, the oral disease remained controlled. Due to the clinical recurrence of Crohn’s disease, Vedolizumab was added as associated treatment, resulting in complete clinical benefit after six months of follow-up. **Conclusions**: This is a unique case of orofacial granulomatosis associated with pan-enteric Crohn’s disease successfully treated with Upadacitinib. More data are needed to explore its potential benefits in this clinical condition.

## 1. Introduction and Clinical Significance

Orofacial granulomatosis (OFG) is a rare group of chronic immune-mediated diseases characterized by granulomatous inflammation of the oral or perioral tissues. This condition can be idiopathic or associated with other diseases such as sarcoidosis, tuberculosis, Melkersson–Rosenthal syndrome, granulomatosis with polyangiitis (GPA), or Crohn’s disease. A delayed hypersensitivity reaction with a predominant Th1-mediated immune response seems to be the etiopathogenic mechanism and leads to the development of non-caseating granulomas, a histological landmark of this disease.

This inflammatory process can lead to several signs and symptoms such as recurrent and persistent lip and buccal mucosal swelling, oral ulcerations, facial palsy, and cervical lymphadenopathy, all associated with general discomfort and significantly impacting the patients’ quality of life [1,2].

The relationship between OFG and Crohn’s disease (CD) is unclear: the oral cavity is a potential site involved, and it is usually more severely affected during active disease. However, up to 30% of patients may present oral lesions despite disease control [3]. Lesions can be specific or nonspecific based on the presence or absence of granuloma at histopathological examination. In addition to oral ulcers, they include face or lip swelling, gingival enlargements, tongue ridges or grooves, cervical lymphadenopathy, and episodes of facial paralysis, which often compromise speaking or eating.

No treatments have been approved to date for the management of OFG. IBD-associated oral manifestations are usually associated with active gut disease so that the main purpose is to control intestinal inflammation. Moreover, effective treatments mainly involve topical or intralesional corticosteroid injections or, in more severe cases, a variety of immunosuppressive treatments including several biologics approved for CD (i.e., Infliximab, Adalimumab, Vedolizumab, or Ustekinumab), though with variable outcomes [2,3,4].

Upadacitinib is a Janus kinase-1 (JAK-1) selective inhibitor, recently approved for the treatment of moderate-to-severe Crohn’s disease, with good efficacy and safety data in both induction (U-EXCEL and U-EXCEED studies) and the maintenance of remission (U-ENDURE study) [5]. Moreover, the drug not only works for symptom resolution but also guarantees a good quality of life [6].

Upadacitinib has also been used in combination with other biologic drugs (i.e., dual-targeted therapy), such as Ustekinumab, in cases of refractory luminal disease or uncontrolled extraintestinal symptoms, with good efficacy and without worrisome safety signals in short or medium time intervals [7,8].

The evidenced role of the Janus kinase–signal transducer and activator of transcription (JAK-STAT) pathway in granuloma formation has brought interest in using JAK inhibitors for granulomatous skin diseases and sarcoidosis [9]. This pathway is in fact used by a variety of inflammatory cytokines, including interferon-Υ, a critical cytokine in granuloma formation, as confirmed by tissue analyses in patients affected by cutaneous sarcoidosis or granuloma annulare. JAK inhibitors are variably selective in their ability to inhibit different parts of the JAK-STAT pathway. In particular, treatment with Tofacinib has been described as effective in downregulating interferon-γ and interleukin-6 messenger RNA in the JAK-STAT pathway [10].

To date, no cases have been reported in the literature on the use of Upadacitinib in OFG, and scarce data exist describing its association with Vedolizumab in IBD.

## 2. Case Presentation

**Figure 1:**
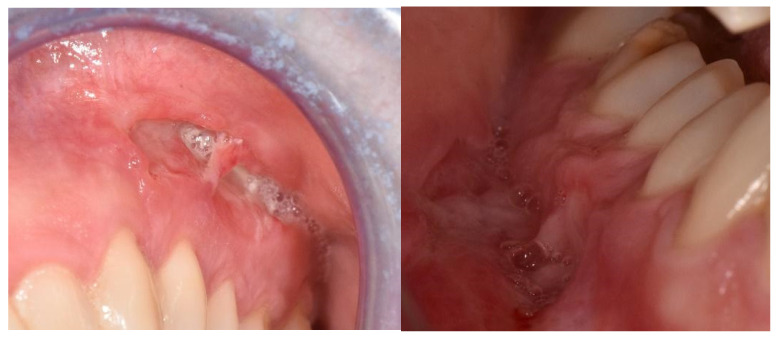
Deep oral ulcers and gingival enlargement as OFG presentation.

We here report the case of a 26-year-old woman with pan-enteric non-stricturing and non-penetrating Crohn’s disease since the age of 10 (Montreal A1, L3 + L4, B1). She was initially treated with 5 mg/Kg Infliximab (IFX) and 1 mg/Kg Azathioprine when, in 2009, she developed buccal swelling and deep and painful oral ulcers (Figure 1) with impaired swallowing. Histological examination describing the presence of chronic granulomatous inflammation confirmed the hypothesis of an associated orofacial granulomatosis (OFG).

The recurrence of oral ulcers along with systemic and intestinal symptoms (i.e., mild fever, asthenia, and abdominal pain) led to the discontinuation of “combo therapy” and the initiation of Thalidomide, which was stopped the following year due to a convulsive episode. Subsequent treatments with Adalimumab and Methotrexate were discontinued due to primary failure as treatment for Crohn’s disease. Furthermore, she did not experience any benefit on oral ulcers.

In 2014, 10 mg/Kg Infliximab was resumed until 2017, when it was stopped again in relation to the suspicion of secondary vasculitis due to the onset of low-grade recurrent fever, mild persistence in C-reactive protein (CRP) increase (up to 1.5 mg/dL), and mild transitory serum creatinine increase (up to 1.6 mg/dL). Due to the lack of a clear diagnosis and the normalization of laboratory indexes, IFX therapy was resumed after the recurrence of intestinal and oral symptoms. It led to the resolution of granulomatosis with a 4-year well-being period. In 2021, two consecutive hospitalizations occurred due to fever (up to 38 degrees Celsius), right-sided pain, hyperchromic urine, and the recurrence of oral ulcers. Antibiotic and corticosteroid therapy with 1500 mg/daily Metronidazole and 60 mg/daily Methylprednisolone was added, without benefit on oral ulcers. Given the positivity for anti-DNA antibodies and the presence of proteinuria, leading to the diagnosis of secondary “lupus-like” connective tissue disorder and immune complex-induced glomerulonephritis, Infliximab was discontinued.

In March 2022, Ustekinumab (390 intravenous induction, followed by 90 mg subcutaneous q8w maintenance), “combo therapy” with 2 mg/Kg Azathioprine was started. However, despite adding topical therapy with Cyclosporine gel and Budesonide daily application, followed by another cycle with systemic steroids (50 mg/daily Prednisone), the benefit on oral ulcers was scarce. In 2023, due to the secondary loss of response to Ustekinumab for Crohn’s disease, accompanied by increased CRP levels (up to 12 mg/dL) in the presence of mild (10.8 g/dL) normocromic anemia, compassionate induction therapy with 45 mg/day Upadacitinib was started with the complete resolution of the oral ulcers at the end of the 12-week induction. The efficacy on oral disease was maintained after the transitioning to a maintenance dosage of 30 mg/day; however, due to the recurrence of Crohn’s disease, following multidisciplinary evaluation, Vedolizumab dual-targeted therapy (DTT) was started, leading to complete clinical benefit. Six months of follow-up showed the persistent remission of the oral disease, without buccal swelling, oral pain, superficial or deep ulcers, or difficulties in swallowing. CRP levels dropped to 1.6 mg/dL. No clinical or biochemical (i.e., increase in lipid levels or blood count changes) side effects of this combination were reported. Endoscopic revaluation has still not been performed, in accordance with the patient’s wishes.

## 3. Discussion

Orofacial granulomatosis (OFG) associated with Crohn’s disease presents a significant therapeutic challenge due to its chronic and recurrent nature and the lack of approved therapies. Various treatments have been described, including topical or systemic corticosteroids, immunosuppressants, and biologic drugs (i.e., Infliximab, Adalimumab, Vedolizumab, and Ustekinumab), with varying degrees of success [2,3,4,11]. In the current case, the patient experienced limited benefit from initial treatments with Infliximab and Azathioprine “combo-therapy”, as well as subsequent therapies including Thalidomide, Adalimumab, Methotrexate, Infliximab monotherapy, and Ustekinumab. The addition of topical steroid and immunosuppressive treatment did not provide additional benefit. The recurrence of oral ulcers and systemic symptoms necessitated multiple changes in therapeutic strategy.

Upadacitinib, a Janus kinase (JAK) inhibitor that selectively inhibits JAK1, modulates the JAK-STAT signaling pathway, which is crucial in the pathogenesis of inflammatory diseases, through the intracellular modulation of the signals of several inflammatory cytokines. This mechanism of action has shown efficacy in controlling inflammation, providing symptomatic relief in various autoimmune conditions, as well as rheumatologic or dermatologic diseases. Upadacitinib has been recently approved to treat adults with moderate-to-severe active CD and for the maintenance of remission [12,13,14].

Evidence suggests that both cytokines and innate immunity play a role in activating the JAK-STAT pathway in granulomatous inflammation, leading to new therapeutic options for refractory diseases. Few studies have described the use of JAK inhibitors such as Tofacitinib, Baricitinib, and Upadacitinib for granulomatous diseases such as disseminated granuloma anulare with positive outcomes [9].

Vedolizumab, an integrin receptor antagonist, specifically targets the gut, reducing inflammation and maintaining remission in Crohn’s disease [15]. It is recommended for the induction of response and remission in patients with moderate-to-severe Crohn’s disease who have had an inadequate response to conventional therapy and/or to anti-TNF therapy [16].

Despite the scarce available evidence, safety issues of dual-targeted therapy involve a moderate proportion of infectious complications among which respiratory and urinary tract infections are the most common ones. No fatal adverse events or serious infections have been described to date [17].

The combination of Upadacitinib with Vedolizumab in our case provided a comprehensive approach, effectively managing both the orofacial and intestinal manifestations of the disease without side effects after 6 months of follow-up.

## 4. Conclusions

Currently, there is a paucity of data on the optimal management of OFG, particularly in the context of Crohn’s disease.

This case highlights the importance of a personalized therapeutic approach in managing complex cases of Crohn’s disease and associated orofacial granulomatosis. The fast effectiveness and good tolerance of JAK inhibitors, particularly Upadacitinib, in OFG are very encouraging. Tailoring treatment to manage both intestinal and extra-intestinal symptoms is essential for achieving optimal patient outcomes. Furthermore, a multidisciplinary approach is crucial for providing comprehensive care and effective disease management. Larger cohort studies or clinical trials are warranted to confirm these findings and to explore the long-term efficacy and safety of such combination therapies.

## Data Availability

Data supporting reported results can be found in Azienda Sanitaria Universitaria Giuliano Isontina (ASUGI) electronic archives.

## Abbreviations

The following abbreviations are used in this manuscript:

IBD	Inflammatory bowel disease
CD	Crohn’s disease
OFG	Orofacial granulomatosis
JAK	Janus kinase
DTT	Dual-targeted therapy
CRP	C-reactive protein
GPA	Granulomatosis with polyangiitis
IFX	Infliximab
